# Case report: A unified CTA–fluoroscopy roadmap for single-session pulsed field ablation and left atrial appendage occlusion

**DOI:** 10.3389/fcvm.2026.1930811

**Published:** 2026-09-22

**Authors:** Guoping Chen, Feiyu Li, Zeqiang Li, Yu Gao, Nan Chang, Bo Liu, Yushun Zhang

**Affiliations:** 1 Interventional Center, Xi'an Daxing Hospital, Xi'an, China; 2 Department of Advanced Therapies, Siemens Healthineers Digital Technology (Shanghai) Co., Ltd., Shanghai, China; 3 Department of Advanced Therapies, Siemens Healthineers Ltd., Shanghai, China; 4 Department of Cardiology, Xi'an Daxing Hospital, Xi'an, China; 5 Department of Cardiovascular Surgery, The First Affiliated Hospital of Xi'an Jiaotong University, Xi'an, China

**Keywords:** atrial fibrillation, computed tomography angiography, image fusion, left atrial appendage occlusion, pulsed field ablation

## Abstract

Single-session pulsed field ablation (PFA) and left atrial appendage occlusion (LAAO) is a clinically attractive strategy for selected patients with atrial fibrillation who require both rhythm-control therapy and stroke-prevention intervention. However, the combined procedure requires the operator to maintain spatial orientation while moving from transseptal access to pulmonary vein targeting and then to appendage engagement and device deployment. In this retrospective, single-centre technical case series, we evaluated five consecutive patients undergoing single-session PFA and LAAO with computed tomography angiography (CTA)–fluoroscopy fusion as a unified anatomical roadmap. Pre-procedural CTA was registered to intraoperative DynaCT, and a simplified CTA-derived model of the left atrium, pulmonary veins, superior vena cava, interatrial septum, and left atrial appendage was overlaid on live fluoroscopy. The same registered roadmap was intended to be retained across transseptal access, PFA localization, and LAAO deployment. The workflow was fully implemented in four patients and interrupted in one because of workflow-related factors during early adoption. PFA and WATCHMAN implantation were completed in all patients. At 45-day CTA follow-up, no residual leak or device-related thrombus was observed. Quantitative registration error and component-specific image-processing times were not prospectively recorded, and no conventional-guidance comparator was available. This proof-of-concept case series demonstrates the feasibility of translating pre-procedural CTA planning into a retained intraprocedural fluoroscopic roadmap across the electrophysiological and structural phases of a single-session intervention.

## 1 Introduction

Pulsed field ablation (PFA) has become an important non-thermal ablation strategy for atrial fibrillation (AF), with pivotal and real-world studies reporting favourable safety and effectiveness profiles for pulmonary vein isolation (1–4). Left atrial appendage occlusion (LAAO) is an established stroke-prevention option for selected patients with non-valvular AF, particularly when long-term oral anticoagulation is unsuitable or associated with increased bleeding concern (5, 6). For patients who require both rhythm-control therapy and stroke-prevention intervention, single-session PFA and LAAO is clinically attractive because both procedures can be completed through a shared transseptal workflow. Early clinical studies have supported the feasibility of combining PFA and LAAO in one session (7–10).

The combined procedure creates a specific imaging challenge. PFA requires catheter orientation at the pulmonary venous ostia and antra before electrophysiological confirmation of isolation. LAAO requires assessment of appendage morphology, working projection, sheath alignment, device coaxiality, compression, and residual leak. Although both procedures occur within the same left atrium, the operator must transition from pulmonary vein targeting to appendage engagement and device deployment while integrating fluoroscopy, transoesophageal echocardiography, angiography, pre-procedural imaging, and electrophysiological assessment.

This challenge may be particularly relevant in patients with AF-related atrial remodelling. Long-standing AF is associated with progressive structural and functional changes of the left atrium, including atrial enlargement, fibrosis, and altered chamber geometry (11, 12). These changes may make fluoroscopic orientation less intuitive, especially when a single transseptal access must support both pulmonary vein ablation and subsequent appendage closure. Three-dimensional CT reconstruction has been described as a useful adjunct for transseptal puncture planning in patients with variable left atrial anatomy (13).

Cardiac computed tomography angiography (CTA) is widely used for LAAO planning because it provides three-dimensional information on appendage morphology, ostial dimensions, landing zone, depth, thrombus assessment, and adjacent left atrial anatomy (14, 15). CT-derived or reconstructed appendage anatomy can also support selection of optimal fluoroscopic working projections for LAAO (16). CTA–fluoroscopy fusion has been applied to LAAO and other structural heart interventions by projecting patient-specific three-dimensional anatomy onto live fluoroscopy (17–20). However, the use of one retained CTA-derived roadmap across both the electrophysiological and structural phases of single-session PFA and LAAO has not been well described.

We report a five-patient technical case series using CTA–fluoroscopy fusion as a unified anatomical roadmap for single-session PFA and LAAO. The unique focus of this report is not the use of PFA or LAAO alone, but the translation of pre-procedural CTA planning into intraprocedural fluoroscopic guidance across transseptal access, PFA localization, and LAAO deployment.

## 2 Case presentation

### 2.1 Patient information and clinical background

Five consecutive patients underwent planned single-session PFA and LAAO at Xi’an Daxing Hospital. This technical case series follows CARE guidance where applicable. Selection considered AF symptoms, thromboembolic and bleeding risks, suitability for long-term oral anticoagulation, anatomy, and patient preference. Anticoagulation history, treatment limitations, individualized LAAO rationale, and shared decisions are reported in Table 1. Patients 1 and 3 had no absolute anticoagulation contraindication; their preference-sensitive decisions were non-typical and should not be generalized.

**Table 1:** Patient-level timeline, clinical characteristics, procedural workflow, and early outcomes.

Patient	Baseline profile, anticoagulation, and LAAO rationale	Pre-procedural imaging	Intervention and fusion workflow	Procedural metrics^a^	Immediate/30-day outcome	Early follow-up and antithrombotic therapy
1	62-year-old man; paroxysmal AF; palpitation; hypertension, diabetes, and CAD; CHA₂DS₂-VASc 3; HAS-BLED 1. Rivaroxaban 15 mg once daily for 18 months with good adherence; no major bleeding or intolerance. Standard OAC, limited LAAO evidence, and procedural risks were discussed. Strong preference for concomitant device-based prevention supported a non-typical, preference-sensitive indication without an absolute OAC contraindication.	Four PVs; no LAA thrombus; cauliflower LAA; landing zone 22.0 mm; depth 46.6 mm	Fusion used for transseptal access, PFA, and LAAO. Minor clinically usable offset; no repeat registration. 32 PFA applications; WATCHMAN 27 mm.	Procedure time, 92 min; fluoroscopy, 10.7 min; contrast, 57 mL; DAP, 7,037.3 µGy·m^2^; AK, 784 mGy	Acute PVI and LAAO success; 20% compression; no immediate leak or 30-day adverse event. Stay 2 days.	Rivaroxaban 15 mg once daily until the 45-day CTA; thereafter, DAPT through month 6 after implantation, followed by aspirin alone. The 45-day CTA showed no peri-device leak or DRT. ECG at 1 month and 24-hour Holter monitoring at 3 months documented no AF.
2	80-year-old woman; paroxysmal AF; chest tightness/palpitation; hypertension and prior stroke/TIA; CHA₂DS₂-VASc 6; HAS-BLED 3. Rivaroxaban 15 mg once daily for 24 months with good adherence; no major bleeding or intolerance. Advanced age, prior stroke, high bleeding risk, and concern about lifelong OAC supported LAAO after shared decision-making.	Four PVs; no LAA thrombus; chicken-wing LAA; landing zone 22.9 mm; depth 31.3 mm	Fusion used for transseptal access, PFA, and LAAO; qualitative overlay checks were adequate; no repeat registration. 24 PFA applications; WATCHMAN 27 mm.	Procedure time, 112 min; fluoroscopy, 28.5 min; contrast, 85 mL; DAP, 17,427 µGy·m^2^; AK, 2,012 mGy	Acute PVI/LAAO success; 22% compression; no immediate leak. Femoral pseudoaneurysm (28 × 18 mm, day 1) treated by thrombin injection; no transfusion, reintervention, or sequelae. Stay 5 days.	Rivaroxaban 10 mg once daily plus clopidogrel 75 mg once daily until the 45-day CTA; thereafter, DAPT for 3 months, followed by aspirin alone. The 45-day CTA showed no peri-device leak or DRT. ECG at 3 months documented no AF; prolonged rhythm monitoring was not performed.
3	52-year-old woman; paroxysmal AF; palpitation/dyspnoea; hypertension; CHA₂DS₂-VASc 2; HAS-BLED 1. Rivaroxaban 20 mg once daily for 6 months was repeatedly interrupted by anticoagulation-associated heavy menstrual bleeding during perimenopause and iron-deficiency anaemia requiring oral iron. No transfusion, hospitalization, critical-site bleeding, haemoglobin decrease ≥2 g/dL, or ISTH-defined major bleeding occurred. Poor tolerance and informed preference supported a lower-risk, non-typical indication without an absolute OAC contraindication.	Four PVs; no LAA thrombus; chicken-wing LAA; landing zone 23.8 mm; depth 21.3 mm	Registration completed, but intended cross-phase fusion use was interrupted by TEE probe dislodgement, prolonged anaesthesia, operator transition, and limited interface familiarity. No repeat registration. 56 PFA applications; WATCHMAN 31 mm.	Procedure time, 109 min; fluoroscopy, 23.4 min; contrast, 120 mL; DAP, 13,419 µGy·m^2^; AK, 1,887 mGy	Acute PVI and LAAO success; 16% compression; no immediate leak or 30-day adverse event. Stay 5 days.	Rivaroxaban 20 mg once daily until the 45-day CTA; thereafter, DAPT through month 6 after implantation, followed by aspirin alone. The 45-day CTA showed no peri-device leak or DRT. ECG at 1 month and 24-hour Holter monitoring at 3 months documented no AF.
4	72-year-old man; paroxysmal AF; palpitation; hypertension, diabetes, and CAD; CHA₂DS₂-VASc 4; HAS-BLED 3. Rivaroxaban 15 mg once daily for 20 months with good adherence; no major bleeding or intolerance. Uncontrolled hypertension, age >65 years, concomitant antiplatelet therapy for CAD, and preference to avoid lifelong OAC supported LAAO.	Four PVs; no LAA thrombus; windsock LAA; landing zone 31.6 mm; depth 40.4 mm	Fusion used for transseptal access, PFA, and LAAO; qualitative overlay checks were adequate; no repeat registration. 48 PFA applications; WATCHMAN 31 mm.	Procedure time, 84 min; fluoroscopy, 20.5 min; contrast, 60 mL; DAP, 15,261 µGy·m^2^; AK, 959 mGy	Acute PVI and LAAO success; 16% compression; no immediate leak or 30-day adverse event. Stay 3 days.	Rivaroxaban 15 mg once daily until the 45-day CTA; thereafter, DAPT through month 6 after implantation, followed by aspirin alone. The 45-day CTA showed no peri-device leak or DRT. ECGs at 1 and 3 months documented no AF; prolonged rhythm monitoring was not performed.
5	81-year-old woman; paroxysmal AF; palpitation; hypertension; weight 54 kg; CHA₂DS₂-VASc 4; HAS-BLED 2. Apixaban 2.5 mg twice daily for 12 months was on-label for age >80 years and low body weight; doses were frequently missed. No major bleeding occurred. Recurrent falls with head-impact risk and unreliable long-term OAC despite counselling supported LAAO.	Four PVs; no LAA thrombus; cauliflower LAA; landing zone 22.7 mm; depth 43.8 mm	Fusion used for transseptal access, PFA, and LAAO; qualitative overlay checks were adequate; no repeat registration. 53 PFA applications; WATCHMAN 27 mm.	Procedure time, 98 min; fluoroscopy, 18.6 min; contrast, 75 mL; DAP, 15,129 µGy·m^2^; AK, 890.7 mGy	Acute PVI and LAAO success; 14%–18% compression; no immediate leak or 30-day adverse event. Stay 4 days.	Rivaroxaban 15 mg once daily until the 45-day CTA; thereafter, DAPT for 3 months, followed by aspirin alone. The 45-day CTA showed no peri-device leak or DRT. ECG at 1 month and 24-hour Holter monitoring at 3 months documented no AF.

a DAP and AK include the intraoperative DynaCT acquisition. DAPT consisted of aspirin 100 mg once daily plus clopidogrel 75 mg once daily. AF, atrial fibrillation; AK, cumulative air kerma; CAD, coronary artery disease; CTA, computed tomography angiography; DAP, dose-area product; DAPT, dual antiplatelet therapy; DRT, device-related thrombus; ECG, electrocardiogram; LAA, left atrial appendage; LAAO, left atrial appendage occlusion; OAC, oral anticoagulation; PFA, pulsed field ablation; PV, pulmonary vein; PVI, pulmonary vein isolation; TEE, transoesophageal echocardiography; TIA, transient ischaemic attack.

Median age was 72 years, and three patients were female. All patients had paroxysmal AF and hypertension. Median CHA₂DS₂-VASc score was 4, and median HAS-BLED score was 2. No patient had evidence of left atrial appendage thrombus before the procedure. Patient-level clinical characteristics, imaging findings, procedural timeline, fusion-roadmap implementation, and early outcomes are summarized in Table 1.

### 2.2 Diagnostic assessment and pre-procedural imaging

All patients underwent pre-procedural ECG-gated contrast-enhanced cardiac CTA on a third-generation dual-source CT system (SOMATOM Force; Siemens Healthineers, Forchheim, Germany) within the routine pre-procedural planning interval. CTA was performed in the left atrial/arterial phase using prospective ECG triggering when feasible. Tube voltage was selected at 80–100 kVp with automatic tube-current modulation. Iodinated contrast medium, 60–70 mL, was injected through an antecubital vein at 4.5–5.0 mL/s, followed by a 30–40 mL saline flush. Images were reconstructed at 0.6–0.75 mm slice thickness using a standard cardiovascular reconstruction kernel and iterative reconstruction. Delayed LAA imaging was obtained without additional contrast injection when thrombus exclusion required confirmation.

Pre-procedural CTA showed four pulmonary veins in all patients and no LAA thrombus. LAA morphology was cauliflower in two patients, chicken-wing in two, and windsock in one. CTA was used to assess pulmonary venous anatomy, left atrial geometry, appendage morphology, ostial dimensions, landing zone, and appendage depth before the combined procedure.

### 2.3 Therapeutic intervention

The intended imaging-treatment workflow is shown in Figure 1. The CTA DICOM dataset was imported into the angiography workstation connected to a ceiling-mounted angiography system. After induction of general anaesthesia and establishment of controlled ventilation, non-contrast intraoperative DynaCT was acquired using the 6s DynaCT Body protocol during a brief ventilation pause. DynaCT was used as the intraoperative reference volume for CTA-to-DynaCT registration.

**Figure 1:**
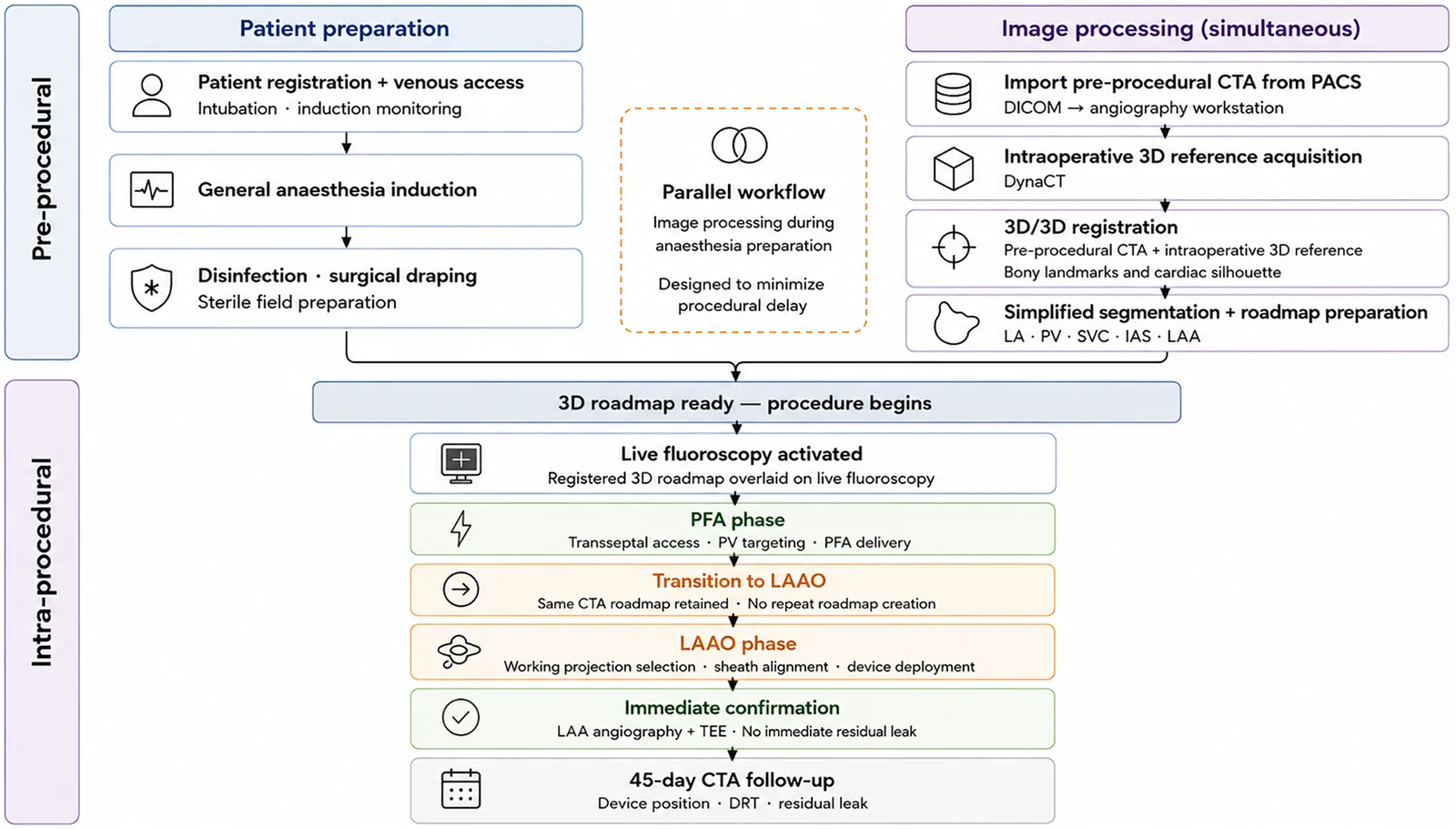
Imaging-treatment workflow for single-session PFA and LAAO using CTA–fluoroscopy fusion. The workflow includes patient preparation, simultaneous image processing, intraoperative three-dimensional reference acquisition, CTA-to-DynaCT registration, simplified roadmap preparation, and activation of the registered roadmap on live fluoroscopy. The same CTA-derived roadmap was intended to be retained across transseptal access, PFA, transition to LAAO, device deployment, immediate confirmation, and early CTA follow-up.

Pre-procedural CTA was registered to intraoperative DynaCT using three-dimensional/three-dimensional registration. Automatic initial alignment was followed by manual fine adjustment in all cases, using concordance of the vertebral bodies, ribs, sternum, cardiac silhouette, and left atrial region. Registration adequacy was assessed qualitatively; no prospectively defined landmark set or quantitative target-registration-error measurement was used. A simplified CTA-derived roadmap was then prepared intraoperatively by an angiography technologist. The workflow included automatic bone removal, manual cropping, and window-width/window-level adjustment. The labelled structures included the left atrium, pulmonary veins, superior vena cava, interatrial septum, and left atrial appendage. The purpose of segmentation was to preserve navigation-relevant anatomy while reducing fluoroscopic clutter. The times required for CTA segmentation, CTA-to-DynaCT registration, and roadmap preparation were not independently time-stamped and could not be reconstructed reliably. CTA-to-DynaCT registration and roadmap generation are shown in Figure 2.

**Figure 2:**
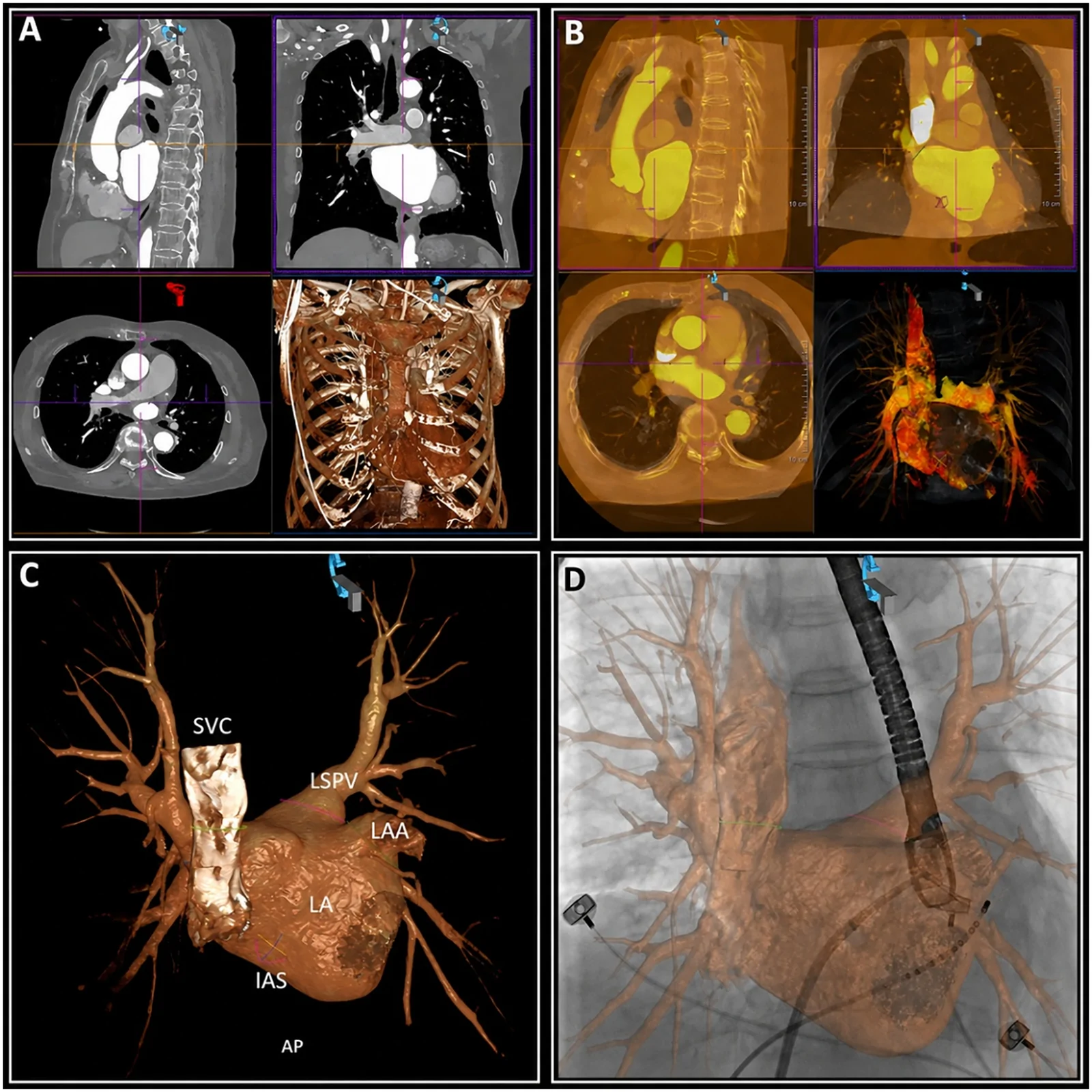
CTA-to-DynaCT registration and roadmap generation. **(A)** Pre-procedural CTA dataset used for assessment of the left atrium, pulmonary veins, superior vena cava, interatrial septum, and left atrial appendage. **(B)** Three-dimensional/three-dimensional registration between pre-procedural CTA and intraoperative DynaCT, using bony landmarks and cardiac silhouette alignment. **(C)** Simplified CTA-derived roadmap after segmentation and roadmap preparation, displaying navigation-relevant left atrial anatomy. **(D)** Registered roadmap overlaid on live fluoroscopy during procedural guidance. The roadmap was used as supplementary anatomical context and did not replace standard fluoroscopic, echocardiographic, angiographic, or electrophysiological assessment.

All procedures were performed under general anaesthesia. Right femoral venous access and transseptal puncture were performed according to standard institutional practice. Transseptal access was guided by fluoroscopy and transoesophageal echocardiography, with the CTA-derived overlay used as supplementary anatomical context for left atrial orientation and interatrial septal spatial assessment.

PFA was performed using the FARAPULSE system (Boston Scientific, Marlborough, MA, USA). During the ablation phase, the CTA-derived roadmap was used to localize the pulmonary venous ostia and antra and to support catheter orientation under fluoroscopy. Acute pulmonary vein isolation was confirmed electrophysiologically. After PFA, the same registered CTA roadmap was retained for LAAO without new CTA import, repeat segmentation, or repeat anatomical registration.

WATCHMAN devices were implanted under fluoroscopic, angiographic, and transoesophageal echocardiographic guidance. During LAAO, the retained roadmap was used to support working projection selection, appendage engagement, sheath orientation, device coaxiality, and spatial assessment during deployment. LAA angiography was matched with the CTA-derived appendage contour to confirm appendage orientation and working projection before device release. Representative overlay use during PFA and LAAO is shown in Figure 3.

**Figure 3:**
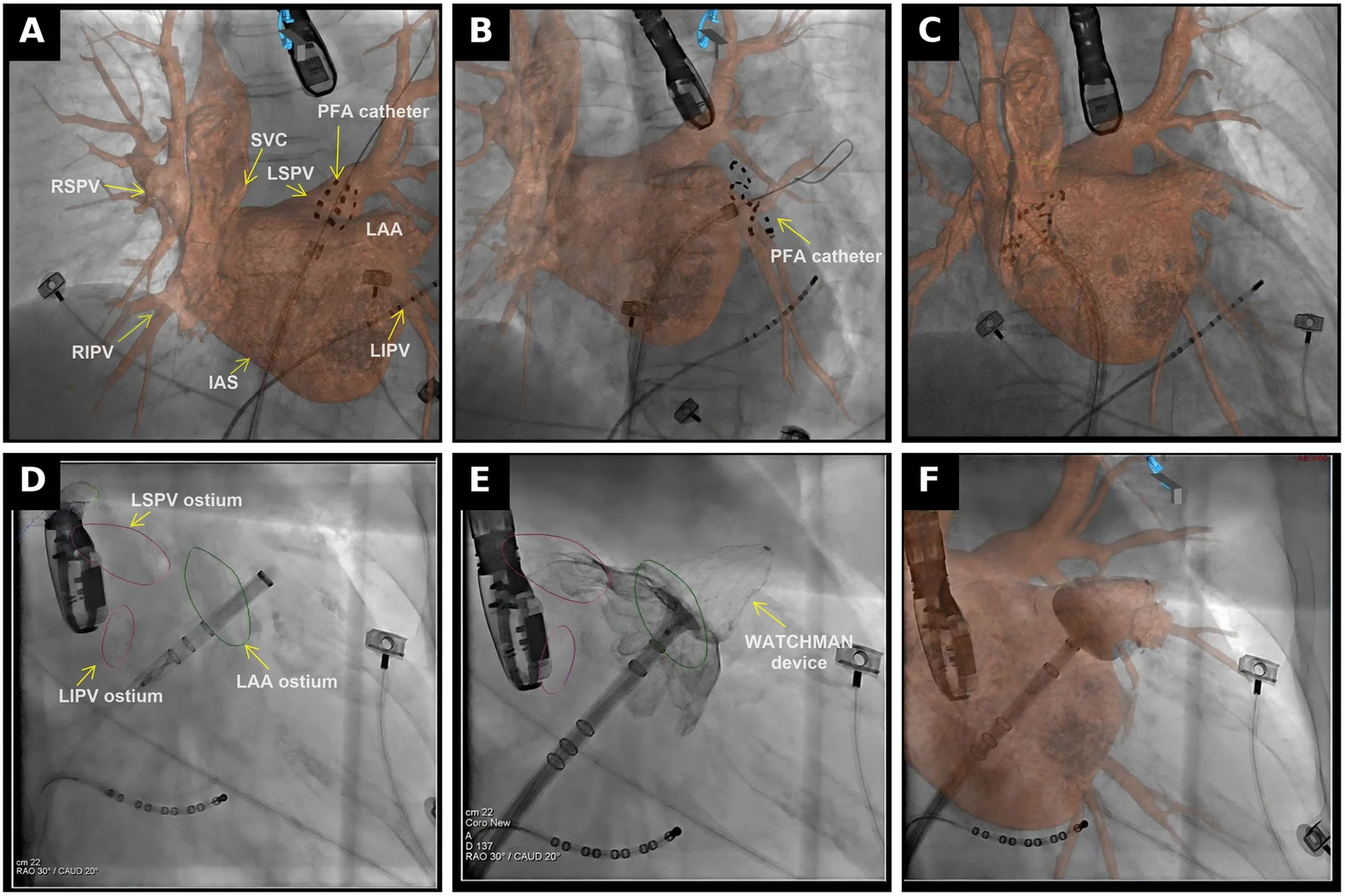
Representative use of the retained CTA-derived roadmap during PFA and LAAO. **(A–C)** The segmented left atrium, pulmonary veins, left atrial appendage, interatrial septum, and superior vena cava were overlaid on live fluoroscopy during PFA. The PFA catheter is shown at representative pulmonary vein targets in different working projections. **(D)** After completion of PFA, the retained roadmap was used for LAAO, with the ostial contours of the LSPV and LIPV shown in magenta and the LAA ostium shown in green. **(E)** LAA angiography and WATCHMAN device deployment were performed with reference to the retained LAA ostial contour. **(F)** Final angiographic assessment after device deployment with the CTA-derived roadmap overlaid. The same registration was maintained throughout the PFA and LAAO phases without repeat segmentation or registration. Yellow arrows and labels indicate the relevant anatomical structures and devices. CTA, computed tomography angiography; IAS, interatrial septum; LAA, left atrial appendage; LAAO, left atrial appendage occlusion; LIPV, left inferior pulmonary vein; LSPV, left superior pulmonary vein; PFA, pulsed field ablation; RIPV, right inferior pulmonary vein; RSPV, right superior pulmonary vein; SVC, superior vena cava.

The fusion overlay was used as an adjunctive roadmap and did not replace standard procedural endpoints. Initial registration was assessed by visual concordance of the vertebral bodies, ribs, sternum, cardiac silhouette, and left atrial region. During transseptal access, PFA, and LAAO, continued overlay adequacy was assessed pragmatically from the relationship between the catheter or sheath and the expected left atrial anatomy, supplemented by transoesophageal echocardiography and angiographic confirmation when applicable. These checks represented qualitative intraprocedural usability rather than quantitative geometric validation. If fusion use was judged impractical or insufficiently reliable, the procedure continued with conventional fluoroscopy, transoesophageal echocardiography, angiography, and electrophysiological assessment according to operator judgement.

The intended CTA–fluoroscopy fusion workflow was fully implemented in four of five patients. In these four patients, the same registered roadmap was used across transseptal access, PFA localization, and LAAO guidance and remained clinically usable without repeat registration. Selective pulmonary venous angiography was not used for PFA localization, and three-dimensional electroanatomical geometry reconstruction was not required for anatomical navigation. These observations describe the implemented workflow and should not be interpreted as evidence of reduced procedure time, contrast use, radiation exposure, or resource utilization.

In one patient, a minor overlay offset was observed but did not preclude roadmap use for catheter orientation and did not require repeat registration. In another patient, fusion guidance was planned but was not carried through the intended procedural sequence because of transoesophageal echocardiography probe dislodgement, prolonged anaesthesia, operator transition, and limited familiarity with the fusion interface during early adoption. This was considered a workflow interruption rather than a failure of CTA import or CTA-to-DynaCT registration.

### 2.4 Follow-up and outcomes

PFA and LAAO were completed in all five patients. Acute pulmonary vein isolation and successful WATCHMAN implantation were achieved in every case. Median number of PFA applications was 48. WATCHMAN device size was 27 mm in three patients and 31 mm in two patients. Final device compression ranged from 14% to 22%, and no immediate residual leak was detected.

Procedure time ranged from 84 to 112 min (median, 98 min), fluoroscopy time from 10.7 to 28.5 min (median, 20.5 min), and contrast volume from 57 to 120 mL (median, 75 mL). Dose-area product ranged from 7,037.3 to 17,427 µGy·m^2^ (median, 15,129 µGy·m^2^), and cumulative air kerma ranged from 784 to 2,012 mGy (median, 959 mGy). Dose-area product and cumulative air kerma were obtained from the angiography-system dose report for the complete procedure and included the intraoperative DynaCT acquisition. Patient-level procedural metrics are provided in Table 1. Median hospital stay was 4 days. One patient developed a 28 × 18 mm femoral pseudoaneurysm on post-procedure day 1; ultrasound-guided thrombin injection achieved complete thrombosis without transfusion, additional intervention, or long-term sequelae. No tamponade, stroke, device embolization, peri-procedural death, or clinically evident device-related thromboembolic event occurred.

At 45-day follow-up, all five patients underwent routine clinical CTA, which showed no peri-device leak or device-related thrombus. Rhythm follow-up comprised 12-lead ECGs in all patients and 24-hour Holter monitoring in patients 1, 3, and 5. No AF was documented, but asymptomatic recurrence cannot be excluded because systematic prolonged monitoring was not performed. Oral anticoagulation was then discontinued, followed by dual antiplatelet therapy and aspirin alone as detailed in Table 1.

## 3 Discussion

This five-patient technical case series illustrates a CTA–fluoroscopy fusion workflow for maintaining anatomical continuity during single-session PFA and LAAO. The main lesson is that one registered CTA-derived roadmap can be retained across transseptal access, pulmonary vein targeting, transition to appendage closure, and device deployment. In four patients, this workflow was implemented across all planned procedural phases without repeat registration. The roadmap was used as an adjunct to fluoroscopy, transoesophageal echocardiography, angiography, and electrophysiological endpoints, not as a substitute for them.

Previous studies have established PFA as an effective non-thermal ablation strategy for pulmonary vein isolation in patients with AF (1–4). However, PFA still requires accurate anatomical orientation at the pulmonary venous ostia and antra. This is particularly relevant in patients with AF-related atrial remodelling, as long-standing AF is associated with left atrial enlargement, fibrosis, and altered atrial geometry (11, 12). Such changes may make fluoroscopic orientation and transseptal access less intuitive, especially when the puncture site must support both ablation and subsequent appendage closure. Three-dimensional CT reconstruction has been described as a useful adjunct for transseptal puncture planning in patients with variable left atrial anatomy (13). In the present series, the CTA-derived roadmap provided additional patient-specific spatial context during transseptal access and early left atrial orientation, while transoesophageal echocardiography remained essential for procedural safety.

Combined PFA and LAAO has been reported as feasible in selected patients (7–10). Those reports mainly focused on procedural completion, short-term safety, and the clinical rationale for addressing rhythm control and stroke prevention in one session. The present series is similar in that all patients underwent planned single-session PFA and LAAO. Its emphasis, however, is different: it focuses on the imaging challenge created by the combined workflow. The operator must move from pulmonary vein localization to appendage engagement, sheath alignment, and device deployment within the same left atrium, often across different fluoroscopic projections and imaging modalities. A retained CTA-derived roadmap may help bridge this transition by preserving one patient-specific anatomical reference throughout the procedure.

Published series provide procedural context. In 10 selected patients, Beney et al. reported median procedure and fluoroscopy times of 79 and 21 min and a median radiation dose of 1,539 cGy·cm^2^, equivalent to 1,539 µGy·m^2^, with no major safety-endpoint event (7). The present medians were 98 min, 20.5 min, and 15,129 µGy·m^2^, respectively; the reported dose–area product was therefore approximately tenfold higher, and one access-site pseudoaneurysm required thrombin injection. Our complete-procedure dose included intraoperative DynaCT, whose independent contribution could not be isolated. Doty et al. reported a prospective 209-patient cohort using a standardized single-transseptal workflow with zero-fluoroscopy PFA, ICE/EAM guidance, and CT-based planning in 69%; mean total procedure time was 57.3 ± 17.0 min and LAAC-segment fluoroscopy time was 3.4 ± 0.8 min (10). This larger study provides contemporary workflow context, not a direct benchmark: case mix, imaging, devices, equipment, acquisition protocols, endpoint definitions, and dose reporting differed. Present-series contrast volume was 75 mL (57–120 mL), but comparable total-procedure contrast data were unavailable. Direct efficiency or safety inference is therefore inappropriate.

The incremental role of the roadmap is therefore most plausibly supplementary rather than substitutive. Potential settings meriting prospective evaluation include markedly remodelled atria, complex pulmonary venous or LAA morphology, a challenging transseptal trajectory that must support both procedural phases, transitions between fluoroscopic projections, and suboptimal echocardiographic visualization. The present series was not designed to determine benefit within any of these subgroups. Accordingly, use of the fusion workflow should be individualized rather than routine, with its anticipated value in maintaining a consistent anatomical roadmap weighed against the overall procedural radiation burden. The anatomical and procedural settings outlined above should be regarded as hypotheses for future prospective evaluation rather than validated patient-selection criteria.

A stable three-dimensional roadmap may also have educational value by providing a shared visual reference for the electrophysiology, structural, echocardiography, radiography, and anaesthesia teams during transitions between procedural phases. It could potentially support anatomical explanation, team communication, and supervised orientation during early operator experience. These educational and learning-curve effects were not formally measured, and the interrupted case indicates that training, role allocation, and interface familiarity remain prerequisites rather than demonstrated benefits.

The approach has several potentially useful workflow characteristics. First, it may support transseptal and early left atrial orientation in patients with remodelled or anatomically variable atria. Second, in fusion-implemented cases, selective pulmonary venous angiography was not used for PFA localization, and three-dimensional electroanatomical geometry reconstruction was not required for anatomical navigation. These observations demonstrate technical feasibility but do not establish lower contrast use, shorter procedure time, lower radiation exposure, or reduced resource use. Third, CTA is already widely used for LAAO planning because it provides three-dimensional information on appendage morphology, ostial dimensions, landing zone, depth, thrombus status, and surrounding left atrial anatomy (14, 15). CT-derived or reconstructed appendage anatomy can also support selection of optimal fluoroscopic working projections for LAAO (16). In this series, the CTA-derived appendage contour and landing-zone orientation were retained on live fluoroscopy to support working projection selection, appendage engagement, sheath alignment, and device coaxiality assessment, while standard angiographic and echocardiographic confirmation remained necessary.

Previous studies of CT–fluoroscopy fusion and computational modelling have demonstrated value for LAAO guidance and structural heart intervention (17–20). The present case series extends this concept to a combined electrophysiological and structural workflow. Once CTA was registered to intraoperative DynaCT, the roadmap remained linked to fluoroscopic geometry during changes in C-arm angulation, table position, magnification, and procedural phase. This allowed the same anatomical frame of reference to remain available from pulmonary vein localization to appendage occlusion. The cross-phase use of a retained roadmap is the main technical contribution of this report.

This report also has limitations. The series was retrospective, single-centre, and included only five patients without a contemporaneous conventional-guidance comparator. Two patients underwent LAAO on a preference-sensitive basis without an absolute contraindication to oral anticoagulation; therefore, this series should not be interpreted as supporting broader or routine LAAO indications. Registration accuracy was assessed by visual alignment and intraprocedural usability rather than prospectively defined landmarks or quantitative millimetre-level error. Concordance with relatively stable thoracic landmarks does not fully account for displacement of dynamic intracardiac structures caused by cardiac motion, respiration, loading conditions, or procedural manipulation. Component-specific times for CTA segmentation, CTA-to-DynaCT registration, and roadmap preparation were not independently recorded. Accordingly, the present series demonstrates feasibility of maintaining a clinically usable roadmap but does not establish geometric accuracy or net procedural efficiency. The workflow was interrupted in one patient because of transoesophageal echocardiography probe dislodgement, prolonged anaesthesia, operator transition, and limited familiarity with the fusion interface during early adoption. This case highlights a key limitation of the approach: fusion guidance depends not only on software capability but also on team coordination, operator familiarity, and workflow stability.

The early outcomes were reassuring, with successful PFA and WATCHMAN implantation in all patients and no residual leak or device-related thrombus at 45-day CTA follow-up. These findings are relevant because peri-device leak and device-related thrombus remain important concerns after LAAO (21–24). However, this case series cannot determine whether CTA–fluoroscopy fusion improves clinical outcomes, reduces radiation exposure, decreases contrast volume, or shortens procedure time. Larger prospective studies are required to evaluate registration error, roadmap preparation time, overlay adjustment, transition time from PFA to LAAO, pulmonary venous angiography use, device recapture, residual leak, device-related thrombus, rhythm outcomes, and operator-rated usability.

Three take-away lessons emerge from this case series. First, in single-session PFA and LAAO, the value of fusion guidance is not merely visualization of individual structures, but preservation of one patient-specific anatomical coordinate system across transseptal access, pulmonary vein ablation, and appendage occlusion. This continuity may be particularly useful when AF-related left atrial remodelling or appendage variability makes fluoroscopic orientation less intuitive. Second, in fusion-implemented cases, pulmonary venous localization was performed without selective pulmonary venous angiography and without separate three-dimensional electroanatomical geometry reconstruction for anatomical navigation; these are observed workflow features rather than evidence of improved efficiency. Third, the same roadmap can translate CTA-based LAAO planning into fluoroscopic execution by supporting working projection selection, appendage engagement, sheath alignment, and device coaxiality assessment during deployment, while standard safety and procedural endpoints remain essential.

## 4 Patient perspective

During routine follow-up, one patient reported that completing PFA and LAAO during the same admission reduced anxiety about needing a second invasive left atrial procedure. The patient felt reassured that rhythm-control therapy and stroke-prevention intervention had been addressed in one procedural course. The pre-procedural explanation of the imaging-guided workflow was also perceived as helpful for understanding the treatment plan.

## Data Availability

The original contributions presented in this article are included in the article. Further inquiries can be directed to the corresponding author, subject to institutional and ethical restrictions.
